# Reperfusion Therapies of Acute Ischemic Stroke: Potentials and Failures

**DOI:** 10.3389/fneur.2014.00215

**Published:** 2014-11-03

**Authors:** Georgios Tsivgoulis, Aristeidis H. Katsanos, Andrei V. Alexandrov

**Affiliations:** ^1^Department of Neurology, The University of Tennessee Health Science Center, Memphis, TN, USA; ^2^Second Department of Neurology, School of Medicine, University of Athens, Attikon University Hospital, Athens, Greece; ^3^International Clinical Research Center, St. Anne’s University Hospital, Brno, Czech Republic; ^4^Department of Neurology, School of Medicine, University of Ioannina, Ioannina, Greece

**Keywords:** reperfusion therapies, stroke, cerebral ischemia, sonothrombolysis, thrombolysis, intravenous, intra-arterial

## Abstract

Over the past 20 years, clinical research has focused on the development of reperfusion therapies for acute ischemic stroke (AIS), which include the use of systemic intravenous thrombolytics (alteplase, desmoteplase, or tenecteplase), the augmentation of systemic intravenous recanalization with ultrasound, the bridging of intravenous with intra-arterial thrombolysis, the use of multi-modal approaches to reperfusion including thrombectomy and thromboaspiration with different available retrievers. Clinical trials testing these acute reperfusion therapies provided novel insight regarding the comparative safety and efficacy, but also raised new questions and further uncertainty on the field. Intravenous alteplase (tPA) remains the fastest and easiest way to initiate acute stroke reperfusion treatment, and should continue to be the first-line treatment for patients with AIS within 4.5 h from onset. The use of tenecteplase instead of tPA and the augmentation of systemic thrombolysis with ultrasound are both novel therapeutical modalities that may emerge as significant options in AIS treatment. Endovascular treatments for AIS are rapidly evolving due to technological advances in catheter-based interventions and are currently emphasizing speed in order to result in timely restoration of perfusion of still-salvageable, infarcted brain tissue, since delayed recanalization of proximal intracranial occlusions has not been associated with improved clinical outcomes. Comprehensive imaging protocols in AIS may enable better patient selection for endovascular interventions and for testing multi-modal combinatory strategies.

## Introduction

Over the last decade, various therapeutic approaches have been developed for treating acute ischemic stroke (AIS) patients presenting with severe neurological deficits (1). As immediate and successful restoration of blood flow in the ischemic tissue was proved to be the paramount target in the treatment of acute cerebral ischemia (2), ongoing clinical research focused on the development of reperfusion therapies for AIS. Reperfusion strategies that were tested in clinical trials include the use of systemic intravenous tPA (3) tenecteplase (4, 5), or desmolteplase (6, 7) the augmentation of systemic tPA-induced recanalization using ultrasound (8, 9), the bridging of intravenous with intra-arterial thrombolysis (10), the use of endovascular multi-modal approaches to reperfusion including mechanical thrombectomy or thromboaspiration using different stent retrievers (11–19) (Tables 1 and 2).

**Table 1:** **Characteristics of acute reperfusion trials in ischemic stroke**.

Study	Type	Centers	Phase	Subgroup	N	Age^a^	Females (%)	Baseline NIHSS^b^	OTT (h)
NINDS (3)	RCT	9	III	IV-tPA	168	69 ± 12.0	43	14	1.5
				Placebo	165	66 ± 13.0	42	15	1.5
TAAIS (5)	RCT	4	IIb	IV-TNK (0.1 mg/kg)	25	72 ± 6.9	48	15 ± 2	3.1
				IV-TNK (0.25 mg/kg)	25	68 ± 9.4	48	15 ± 2	3.0
				IV-tPA	25	70 ± 8.4	52	14 ± 2	2.7
DIAS (6)	RCT	44	II	IV-desmoteplase	75	68	45	12	5.4
				Placebo/SOC	22	68	48	12	5.4
DIAS II (7)	RCT	52	III	IV-desmoteplase (90 mcg/kg)	57	67.9 ± 13.4	53	11 ± 6	6.5
				IV-desmoteplase (125 mcg/kg)	66	70 ± 12.3	56	10 ± 5	6.7
CLOTBUST (8)	RCT	3	II	IV-tPA + TCD	63	67 ± 11.9	35	15	2.5
				IV-tPA	63	70 ± 13.1	52	15 ± 6	2.2
TUCSON (9)	RCT	14	II	IV-tPA + TCD + MB (1.4 ml)	12	63 ± 15.0	54	9 ± 6	2.6
				IV-tPA + TCD + MB (2.8 ml)	11	68 ± 15.0	17	16 (9–21)	2.2
				IV-tPA	12	65 ± 14.0	33	12 (7–14)	2.1
IMS III (10)	RCT	71	III	IA-tPA + MT	434	69 (23–89)	50	17	4.2
				IV-tPA	222	68 (23–84)	45	16	2.0
PROACT II (11)	RCT	54	II	IA-ProUK	121	64 ± 14.0	42	17	4.7
				Placebo	59	64 ± 14.0	39	17	5.1
MERCI (12)	SA	25	II	MT-MERCI	141	67 ± 15.5	46	20 ± 7	4.3
Multi-MERCI (13)	SA	15	II	MT-MERCI	164	68.1 ± 16.0	57	19	4.3
PENUMBRA PST (14)	SA	24	II	MT-penumbra	125	63.5 ± 13.5	49	18 ± 5	4.3
PENUMBRA POST (15)	SA	7	IV	MT-penumbra	157	65 ± 15.0	46	16 ± 6	4.5
SWIFT (16)	RCT	18	II	MT-solitaire	58	67.1 ± 12.0	52	17 ± 5	4.9
				MT-MERCI	55	67.1 ± 11.0	49	18 ± 5	5.3
TREVO 2 (17)	RCT	7	III	MT-Trevo	88	67.4 ± 13.9	55	18 ± 5	4.6
				MT-MERCI	90	67 ± 14.7	60	18 ± 5	4.5
MR-Rescue (18)	RCT	22	IIb	MT (+IV-tPA)(+IA-tPA)-penumbral	34	66.4 ± 13.2	43	16 (11–18)	6.0
				MT (+ IV-tPA)(+ IA-tPA)-non-penumbral	30	61.6 ± 12.0	57	21 (17–22)	6.0
				IV-tPA/SOC-penumbral	34	65.8 ± 16.9	56	16 (11–18)	5.8
				IV-tPA/SOC-non-penumbral	20	69.4 ± 15.9	40	21 (17–23)	5.7
Synthesis expansion (19)	RCT	24	III	IA-tPA + MT	181	66 ± 11.9	41	13 (9–17)	3.8
				IV-tPA	181	67 ± 11.9	43	13 (9–18)	2.8

*N, number of patients; RCT, randomized clinical trial; SA, single-arm study; OTT, onset-to-treatment time; NIHSS, National Institute of Health Stroke Scale; h, hours; TCD, transcranial Doppler; IV, intravenous; tPA, alteplase; TNK, tenecteplase; UK, urokinase; IA, intra-arterial; MT, mechanical thrombectomy; MB, microbubbles; SOC, standard of care. The values that are presented solely without corresponding SDs or IQRs, is because these data were not provided from the original publications. ^a^numbers in parentheses indicate the corresponding range; ^b^numbers in parentheses indicate the corresponding interquartile range (IQR)*.

**Table 2:** **Safety and efficacy outcomes of acute reperfusion trials in ischemic stroke**.

Study	Subgroup	90 d mRS = 0–1 (%)	90 d mRS = 0–2 (%)	Mortality (%)	sICH (%)	Successful recanalization
NINDS (3)	IV-tPA	39	–	17	6.4	–
	Placebo	26	–	21	0.6	–
TAAIS (5)	IV-TNK (0.1 mg/kg)	54	64	8	4	36
	IV-TNK (0.25 mg/kg)	54	64	8	0	35
	IV-tPA	40	44	12	4	80
DIAS (6)	IV-desmoteplase	–	39	4	12	49.3
	Placebo/SOC	–	22	3	0	19.2
DIAS II (7)	IV-desmoteplase (90 mcg/kg)	–	54	5	3.5	–
	IV-desmoteplase (125 mcg/kg)	–	48	21	4.5	–
		–	57	6	0	–
CLOTBUST (8)	IV-tPA + TCD	42	51	15	4.8	49
	IV-tPA	29	37	18	4.8	30
TUCSON (9)	IV-tPA + TCD + MB (1.4 ml)	75	83	9	0	67
	IV-tPA + TCD + MB (2.8 ml)	50	60	30	27	46
	IV-tPA	36	55	0	0	33
IMS III (10)	IA-tPA + MT	29	43	19.1	6.2	ICA:81; M1-MCA: 86; M2-MCA:88; BA: 100
	IV-tPA	27	40	21.6	5.9	ICA: 35; M1-MCA: 68; M2-MCA: 77
PROACT II (11)	IA-ProUK	26	40	25	10	–
	Placebo	17	25	27	4	–
MERCI (12)	MT-MERCI	–	28	8	44	48
Multi-MERCI (13)	MT-MERCI	–	36	34	9.8	69.5
PENUMBRA PST (14)	MT-penumbra	–	25	32.8	11.2	81.6
PENUMBRA POST (15)	MT-penumbra	–	41	20	6	87
SWIFT (16)	MT-solitaire	26	28	17	2	61
	MT-MERCI	18	27	38	11	24
TREVO 2 (17)	MT-Trevo	27	40	34	–	86
	MT-MERCI	15	22	24	–	60
MR-Rescue (18)	MT (+ IV-tPA)(+ IA-tPA)-penumbral	9	14	16	9	77
	MT (+ IV-tPA)(+ IA-tPA)-non-penumbral	6	10	23	0	62
	IV-tPA/SOC-penumbral	15	23	9	6	–
	IV-tPA/SOC-non-penumbral	6	10	22	0	–
Synthesis expansion (19)	IA-tPA + MT	30	42	14	6	–
	IV-tPA	35	31	10	6	–

*mRS, modified Ranking Scale; sICH, symptomatic intracerebral hemorrhage; TCD, transcranial Doppler; IV, intravenous; tPA, alteplase; TNK, tenecteplase; UK, urokinase; IA, intra-arterial; MT, mechanical thrombectomy; MB, microbubbles; SOC, standard of care*.

In the present narrative review, we summarize the evolution of systemic intravenous and endovascular reperfusion strategies for AIS treatment, highlight the comparative safety and efficacy of these modalities, and underscore their potentials and limitations and comment on the future directions of acute reperfusion therapies in acute cerebral ischemia.

## Systemic Reperfusion Therapies

### Intravenous fibrinogen-depleting agents

Fibrinogen-depleting agents may help the restoration of blood flow after an ischemic stroke by reducing the fibrinogen in blood plasma (20). A continuous 72-h intravenous infusion of the defibrinogenerating agent Ancrod beginning within 3 h of stroke onset, followed by infusions lasting approximately 1 h at 96 and 120 h was found to have a favorable benefit–risk profile for patients with AIS in The Stroke Treatment with Ancrod Trial (STAT). The proportion of severely disabled patients was less in the ancrod group than in the placebo group, and this observation was independent from age, stroke severity, sex, prestroke disability, and time to treatment. Both mortality and symptomatic intracerebral hemorrhage rates were not found to be different between treatment groups. However, patients randomized to ancrod were found to have more frequently asymptomatic intracranial hemorrhages compared to placebo (21). In both the subsequent European STAT and the Ancrod Stroke Program, functional 3-month outcome was not found to be significantly different between patients randomized to ancrod or placebo within 6 h of stroke onset (22).

In a meta-analysis of randomized trials, the use of fibrinogen-depleting agents within 14 days of stroke onset was marginally related with a decreased risk of unfavorable outcome and stroke recurrence at follow-up, but with a double risk of symptomatic intracranial hemorrhage (23).

### Thrombolysis with streptokinase

Both the Multicentre Acute Stroke Trial-Italy (MAST-I) and Multicenter Acute Stroke Trial-Europe (MAST-E) resulted that streptokinase cannot be recommended in AIS, as intravenous infusion of 1.5 MU streptokinase (1.5 MU for 1 h) within 6 h of stroke onset was associated with a significantly higher 10-day mortality compared to 300 mg/day buffered aspirin (24, 25). However, data from the Australian Streptokinase (ASK) Trial Study Group suggested that 1.5 MU intravenous streptokinase for 1 h within 3 h of stroke was safer and associated with significantly better outcomes than later treatment, as poor outcomes were confined to patients receiving therapy more than 3 h after stroke onset (26).

### Thrombolysis with tissue plasminogen activator

Intravenous thrombolysis with tissue plasminogen activator (tPA) remains the only evidence-based treatment for AIS and is the fastest and easiest way to initiate acute reperfusion treatment with a 24/7 availability even in primary stroke centers (3). Selection for this treatment remains simple and expeditious requiring only the presence of a disabling neurological deficit, bleeding exclusion (with non-contrast head CT scan), and determination of time from symptom onset (27, 28). However, only a minority of stroke patients arrive in time to be eligible for this treatment (29).

To date, patients with wake-up ischemic stroke are excluded from intravenous thrombolysis *per se* in daily clinical practice, even if they meet all other criteria for treatment. However, emerging data suggest that intravenous thrombolysis in carefully selected wake-up stroke patients, showing no or early ischemic changes on brain imaging, might be safe and effective comparable to those patients with known time of symptom onset (30, 31). Apart from intravenous thrombolysis trials, wake-up stroke patients could also be excellent candidates for future endovascular stroke trials.

The dose of tPA is calculated using patient’s weight and not based on thrombus weight and the success of the treatment is independent from the presence of residual flow that exposes shallow layers of thrombus to circulating tPA (32). In most cases, intravenous tPA induces partial recanalization, since stroke patients often have large thrombus burden (33). Thus, the presence of a proximal arterial occlusion does not necessarily lead to tPA failure, since some degree of recanalization can occur even with large thrombi (33, 34).

Time matters in the clinical outcome of AIS not only as the time from symptom onset to the initiation of treatment but also in the speed of thrombolysis once the tPA infusion is initiated (35–37). With the latest advances in extending the timeframe for IVT (to 4.5 h from symptom onset) (27, 28) and improvement in imaging modalities (to select appropriate candidates for acute reperfusion therapies), it becomes apparent that the efficacy of systemic thrombolysis is time-dependent and as onset-to-treatment time increases the percentage of AIS patients with potentially salvageable infarcted brain tissue decreases (38–40). The finding that shorter time to treatment translates into faster recanalization and better clinical recovery has also been suggested by a meta-analysis of studies that evaluated recanalization for stroke treatment (41). This hypothesis is currently investigated by prospectively acquired data from the ongoing CLOTBUST-PRO (PROspective multi-national CLOTBUST collaboration on reperfusion therapies for AIS) observational study (42).

### Intravenous thrombolysis with tenecteplase

The high rates of unsuccessful reperfusion observed with tPA in patients with large thrombus burden (clots located at terminal internal carotid artery, tandem extracranial internal carotid artery/middle cerebral artery occlusions) triggered the search for other thrombolytic agents in AIS (43, 44). Tenecteplase (TNK) is a genetically engineered variant of tPA that has a longer half-life and is more fibrin specific than tPA. These properties make TNK a very advantageous thrombolytic to induce faster and more complete clot lysis, with less bleeding complications and early re-occlusions (45). An additional benefit is that TNK can be administered by a single intravenous bolus without the need of infusion (46). Even though TNK has already been approved for the treatment of acute myocardial infarction, the use of TNK instead of tPA in the treatment of AIS outside of the setting of a clinical trial still remains unapproved (46, 47).

Only two randomized clinical trials (phase IIB) comparing tPA to TNK in acute stroke patients have been published to date. In the study conducted by the Tenecteplase in Stroke Investigators different tenecteplase doses (0.1, 0.25, and 0.4 mg/kg) were compared with standard 0.9 mg/kg rt-PA dose in patients with acute stroke within 3 h of onset to establish the optimal TNK dose for the subsequent phase III trial. The trial was prematurely terminated for slow enrollment, as between 2006 and 2008 only 112 patients had been randomized at 8 clinical centers. No statistically significant differences were found in the 3-month outcome between the TNK and tPA groups, while symptomatic intracranial hemorrhage rates were found to be highest in the 0.4 mg/kg tenecteplase group and lowest in the 0.1 mg/kg tenecteplase group (4). In the Tenecteplase versus Alteplase for Acute Ischemic Stroke (TAAIS) trial 75 patients, who arrived <6 h after the onset of ischemic stroke with a perfusion lesion at least 20% greater than the infarct core on CT perfusion imaging at baseline and an associated vessel occlusion on CT angiography, were randomly assigned to receive either tPA (0.9 mg/kg) or TNK (0.1 mg/kg or 0.25 mg/kg). Both TNK groups were found to have greater reperfusion rates and better clinical improvement at 24 h than the tPA group, while no significant differences in intracranial bleeding or other serious adverse events were noted between the groups. Additionally, the higher dose of TNK (0.25 mg/kg) was found to be superior to both the lower TNK dose of 0.1 mg/kg and to tPA for all efficacy outcomes, including the 3-month functional outcome (5). In a comprehensive benefit–risk analysis, TAAIS was found to have the higher benefit-to-risk ratio among all acute reperfusion therapies for AIS, followed by sonothrombolysis trials (48). However, it should be noted that the inclusion criteria of TAAIS in imaging greatly favored the selection of patients most likely to benefit from thrombolytic therapy.

### Intravenous thrombolysis with desmoteplase

Because of its high fibrin specificity, non-activation by β-amyloid, long ER terminal half-life and absence of neurotoxicity, desmoteplase is an attractive alternative to alteplase for systemic thrombolytic treatment of AIS (49, 50).

The desmoteplase in acute stroke (DIAS) trial was a safety and dose-finding phase IIb trial in AIS patients with a perfusion-diffusion mismatch on MRI within 3–9 h of symptom onset (6). Initially, patients were randomized to intravenous desmoteplase 25, 37.5, or 50 mg versus placebo. However, the rate of symptomatic intracranial hemorrhage was high in the desmoteplase arm and the study team decided to reduce the doses on the basis of body weight (62.5, 90, and 125 μg/kg). In turn, it was concluded that clinical improvement was associated with early reperfusion. Overall, 54.3% patients treated in 3–6 h and 40% in 6–9 h time window showed reperfusion that was significantly correlated with favorable clinical outcome. Subsequently, a randomized, placebo-controlled, phase III trial (DIAS-2) enrolled AIS patients presenting within 3–9 h of onset with tissue at risk (at least 20% ischemic-penumbra brain tissue evaluated using MRI or CT mismatch) (7). DIAS II investigated the efficacy and safety of two doses of desmoteplase (90 and 125 μg/kg) that were administered as an i.v. bolus. The median baseline NIHSS score was nine points and only 30% of the recruited patients demonstrated a visible arterial occlusion at presentation. Hence, the median core lesion and mismatch volumes were small (10.6 cm^3^ and 52.5, respectively). There was no significant difference in the three groups according to the improvement on the NIHSS score. Mortality was 6.3% in the placebo group, 5.3% in the 90 μg/kg group, and 21.2% in the 125 μg/kg group. SICH was noted in 0, 3.5, and 4.5% of patients in the three treatment arms, respectively. The neutral results of DIAS-2 trial may be attributed to the low baseline NIHSS-scores, the low rates of proximal intracranial occlusions and small absolute mismatch volumes. However, it should be noted that when the DIAS-2 data were analyzed with a more conservative definition of mismatch (PWI-DWI volume >75 mL), beneficial effects of desmoteplase were apparent in comparison to placebo (7). The safety and efficacy of intravenous desmoteplase in AIS patients is currently investigated in two ongoing parallel trials (DIAS-3 and DIAS-4), enrolling patients within a 9 h window and using updated imaging criteria for selection of patients with target mismatch.

## Sonothrombolysis

Since intravenous tPA induces mostly partial recanalization of large thrombi and delivery of tPA to the binding sites in the thrombus relies on often diminished residual blood flow, early augmentation of fibrinolysis to improve arterial recanalization is desirable (32, 34). Ultrasound-induced mechanical agitation of thrombus-residual flow interface can expose shallow layers of thrombus to circulating tPA and facilitate streaming of plasma through thrombus, thus bringing more tPA to binding cites (51–54). Experimental *in vitro* and *in vivo* data suggested that pressure waves from ultrasound can travel through tissues and deliver this mechanical momentum to stagnant flow areas and thrombus interfaces (51, 52).

Sonothrombolysis is the ultrasound targeting of an arterial occlusive clot in order to accelerate the thrombolytic effect of systemic tPA. Mechanical pressure waves, produced by 2 MHz frequency ultrasound energy, can improve the delivery and penetration of the thrombolytic drug inside the clot (55, 56). The first properly powered multicenter clinical trial that confirmed existence of ultrasound-enhanced thrombolysis in human subjects was the Combined Lysis of Thrombus in Brain ischemia using transcranial Ultrasound and Systemic TPA (CLOTBUST) trial. In this trial, patients with acute middle cerebral artery occlusions that were randomized to the combination of intravenous thrombolysis with 2-h continuous transcranial Doppler monitoring were found to achieve higher rates of recanalization within 2 h of treatment (without having higher rates of symptomatic intracerebral hemorrhage) when compared to patients treated only with intravenous tPA (8). A meta-analysis of six randomized and three non-randomized clinical studies of sonothrombolysis indicated that sonothrombolysis with high-frequency ultrasound almost triples the likelihood of complete recanalization and doubles the odds of favorable functional outcome, at no increase in the risk of symptomatic intracerebral hemorrhage, in comparison to standard intravenous thrombolysis (57). Similarly, two other independent meta-analyses have also confirmed that sonothrombolysis appears to reduce death or dependency at 3 months and to increase recanalization, without further augmenting the risk of symptomatic intracranial hemorrhage (58, 59).

Enhancement of tPA-induced recanalization can also be accomplished using microsphere-potentiated ultrasound-enhanced thrombolysis combining high-frequency ultrasound with gaseous microspheres (60–62). These intravenously injected microspheres are able to cross the lung barrier due to their size and stability and undergo expansion in size followed by transient oscillation or complete break up, when intercepted intracranially by an ultrasound beam aimed at thrombus-residual flow interface (63–65). With this mechanism, energy is transmitted to areas with stagnant flow and penetration of tPA into the thrombus is further promoted (66, 67).

Molina et al. pioneered the use of gaseous microspheres in combination with CLOTBUST monitoring methods and reported safety and recanalization rates of microsphere-potentiated sonothrombolysis in a pilot phase IIb randomized-controlled trial (9). The Transcranial Ultrasound in Clinical SONothrombolysis trial (TUCSON) showed that a 1.4 mL dose of perflutren-lipid microspheres do not augment further the risk of symptomatic intracerebral hemorrhage, and thus can be safely co-administered during tPA infusion (9, 68). The combination of perflutren-lipid microspheres, TCD and tPA was associated with 50–67% recanalizations rates of proximal intracranial arterial occlusions (9, 68).

The main limitation of current sonothrombolysis technology (applied with or without microspheres) remains operator-dependency. Skilled sonographers able to perform transcranial Doppler or duplex are largely unavailable to the emergency departments outside a few centers worldwide (69–71). An operator-independent 2-MHz transcranial Doppler device, developed to provide therapeutic ultrasound regardless of sonography skills, is currently being tested in a pivotal, multicenter, phase III randomized-controlled trial, given the preliminary promising results in small phase IIa/IIb safety studies (72, 73).

## Endovascular Reperfusion Therapies

### Intra-arterial thrombolysis

The majority of AIS patients arrive beyond the recommended time window for the administration of intravenous thrombolysis. Moreover, a substantial number of patients with large clot burden (terminal internal carotid artery occlusions, tandem internal carotid artery/middle cerebral artery occlusions) that undergo intravenous thrombolysis will experience no recanalization or develop reocclusion (74, 75). Because persistence of a proximal arterial occlusion has long been recognized as a poor prognostic sign (2), the aforementioned considerations prompted continuing development of catheter-based interventions in order to increase reperfusion rates in AIS patients.

Catheters suitable for navigation into brain vasculature opened the possibility of intra-arterial revascularization with a drug topically delivered to intracranial thrombus. The Prolyse in Acute Cerebral Thromboembolism (PROACT) trial was the first controlled clinical trial that extended the timeframe up to 6 h from symptom onset in patients with proximal middle cerebral artery occlusions (76). This trial showed that intra-arterial pro-urokinase administration was associated with an absolute increase of 15% in good clinical outcomes at 3-month assessment compared to placebo, but also reported a 10% incidence of intracranial hemorrhagic complications with interventions. PROACT investigators also reported an overall recanalization rate of 66% at the completion of intra-arterial treatment (11). In a meta-analysis of randomized clinical trials, intra-arterial thrombolysis with either urokinase or recombinant pro-urokinase administered up to 6 h after ischemic stroke was found to increase significantly the proportion of 3-month favorable outcome, but with a significant increase in symptomatic intracranial hemorrhage within 24 h of treatment (77). Despite these highly encouraging results, the US Food and Drug Administration (FDA) did not approve pro-urokinase as an alternative treatment for stroke and required further efficacy trials.

### Combined endovascular therapies

In parallel with the primary intra-arterial approach development, the group of Interventional Management of Stroke (IMS) investigators started to explore “bridging” therapy, with the initiation of treatment with intravenous tPA followed by intra-arterial tPA infusion if occlusion persisted beyond the duration of systemic treatment (78, 79). The phase III IMS trial compared standard intravenous dose of tPA (0.9 mg/kg) over 1 h infusion to 0.6 mg/kg intravenous tPA over 30 min infusion followed by intra-arterial administration of tPA directly to the intracranial thrombus (80). In April 2012, after 656 subjects were randomized, the study was terminated by the National Institute of Neurological Disorders and Stroke (NINDS) due to conditional power criteria (81). Up to this point, the trial showed similar safety outcomes and no significant difference in functional independence between endovascular therapy after intravenous tPA and intravenous tPA alone (10). However, significant differences were identified between treatment arms for 24-h recanalization in proximal occlusions, as carotid T- or L-type and tandem ICA and M1 occlusions showed greater recanalization rates and a trend toward better outcome with endovascular treatment (82). Interestingly, in a prespecified analysis of IMS III trial, Khatri et al. concluded that every 30-min delay in angiographic reperfusion reduced the relative likelihood of a favorable functional outcome at 3 months by 15% in unadjusted analysis and 12% in adjusted analysis (83). The observations of the IMS investigators (83) in combination with the findings of pooled analyses of intravenous thrombolysis randomized-controlled trials (37) introduce the hypothesis that the time-dependency of the clinical efficacy of acute reperfusion therapies (both systemic and endovascular) in AIS may be attributed to the time-dependency of recanalization of proximal intracranial occlusions independent of the selected therapeutic strategy (84).

In the randomized SYNTHESIS Expansion trial, 362 patients with AIS within 4.5 h after onset were randomly assigned to either intravenous tPA or endovascular therapy (intra-arterial thrombolysis with tPA, mechanical clot disruption or retrieval, or a combination of these approaches). No significant differences were found in both safety (rates of intracranial hemorrhage and death) and long-term outcomes between the two treatment arms. However, the median time from stroke onset to the start of treatment was significantly higher (*p* < 0.001) for the endovascular therapies (3.75 h) compared to intravenous t-PA (2.75 h). Of the 181 patients in the endovascular treatment arm, intra-arterial thrombolysis with a median dose of 40 mg tPA was administered in 109 patients, a device (SOLITAIRE, PENUMBRA, TREVO, or MERCI) was added in 56 patients, while 15 patients did not receive finally any treatment (19).

### Mechanical thrombectomy and aspiration

Currently, the use of intra-arterial revascularization is performed by skilled operators in centers with stroke-trained interventionalists (interventional neuro-radiologists, endovascular neurologists, or neurosurgeons), and thus procedural success is highly dependent on interventionalists’ skills and on the device that is used. Mechanical thrombectomy emerged in stroke with the development of a device called the MERCI retriever. The device deploys a screw-like wire, which engages the thrombus and tracks it toward proximal vessels with larger diameter, and finally aspirates the retrieved parts of a thrombus. The initial studies (MERCI and Multi-MERCI) showed recanalization rates of 46–57% and symptomatic intracranial hemorrhage rates of 7.8–9.8% (12, 13). In the cases of Multi-MERCI clinical study, when an adjunctive therapy, such as intra-arterial injection of a lytic drug, was used, recanalization rate increased to 69.5% (13). Even though in both MERCI and Multi-MERCI studies concurrent controls were lacking and the recanalization rates that they reported were less of that in PROACT trial, FDA Medical Devices panel granted approval for clinical use of MERCI retriever in stroke patients (85). This decision was heavily criticized by many researchers, as the spreading use of this device in routine clinical practice further hindered the ability to test new stroke treatments with proper controls (85).

Another device that received FDA approval after being tested in phase I–II clinical studies without concurrent controls is the PENUMBRA device, a device which aims to aspirate the thrombus from within the vessel (86). The Penumbra Pivotal Stroke Trial enrolled 125 stroke patients presented within 8 h of symptom onset, who had NIHSS ≥8 and an angiographic occlusion of a treatable large intracranial vessel. 81.6% of the treated vessels were successfully revascularized, while procedural events were reported in 16 patients (12.8%) (14). The post-market experience of the PENUMBRA system, consisting of a retrospective case review of 157 consecutive patients treated at 7 international centers, reported revascularization rate, and safety profile of the device comparable to those reported in the Pivotal trial, and a higher proportion of patients who had good functional outcome at 90 days assessment (15). Nevertheless, a *post hoc* analysis comparing the findings of PENUMBRA pivotal trial to the control arm (standard intravenous thrombolysis) of two sonothrombolysis trials (8, 9) reported that despite lower revascularization rates, patients treated with systemic thrombolysis achieved better functional outcomes likely due to earlier treatment initiation (87). These data indicate that it is unrealistic to expect primary intra-arterial revascularization to be any better than systemic plasminogen activator within the 3-h time window. Improvements in the speed of delivery and performance of intra-arterial reperfusion are needed.

The MERCI retriever was compared in two randomized trials with another 2 stent retrievers: the Solitaire Flow Restoration (FR) device, in the SOLITAIRE With the Intention For Thrombectomy (SWIFT) trial and the TREVO, in the Thrombectomy Revascularization of Large Vessel Occlusions in AIS (TREVO 2) trial. In the SWIFT trial, 113 AIS patients with moderate to severe neurological deficits and within 8 h of stroke symptom onset were randomized to receive thrombectomy treatment with either SOLITAIRE or MERCI. The SOLITAIRE FR was found to have substantially better angiographic, safety and clinical outcomes than the MERCI Retrieval System. An almost twofold magnitude of difference, in favor of the SOLITAIRE FR, was observed in good neurological outcome at 3 months. Additionally, mortality rates were found to be lower in the Solitaire group than in the MERCI group (17 and 38%, respectively) (16). Data from a non-randomized study protocol found that stent based thrombectomy with Solitaire AB device was associated with more favorable functional outcome in patients with proximal middle cerebral artery occlusion, when compared to intravenous tPA (88).

TREVO 2 was an open-label randomized-controlled trial that recruited adult stroke patients within 8 h of symptom onset and with angiographically confirmed large vessel occlusion. Patients were assigned to stratified randomization (by age and NIHSS score) to either thrombectomy with TREVO or MERCI devices. The TREVO retriever was found to be superior to the MERCI retriever regarding the efficacy, but the incidence of the procedure-related adverse events did not differ significantly between groups (15% of the patients in the Trevo group and 23% of the patients in the Merci group) (17).

In a systematic review of available studies, mechanical thrombectomy in AIS with a Solitaire stent was found to be both effective and safe, with high recanalization rates (66.7–100%) and low procedural complications (3.4%) (89). On the basis of the aforementioned results, the FDA granted the approval of both SOLITAIRE FR and TREVO stent-retriever devices for the treatment of AIS due to large vessel occlusion. In view of the higher efficacy (in terms of recanalization) and safety (in terms of periprocedural complications), the current AHA guidelines underscore that stent retrievers such as SOLITAIRE FR and TREVO are generally preferred to coil retrievers such as MERCI, when mechanical thrombectomy is pursued (Class I; Level of Evidence A), while the relative effectiveness of the PENUMBRA System versus stent retrievers is not yet characterized (27).

The MR RESCUE trial tested the hypothesis that a favorable penumbral pattern (substantial salvageable tissue and small infarct core) on CT or MRI scan can identify stroke patients that are more likely to benefit from acute endovascular treatment. Patients with confirmed large vessel occlusion were randomized to receive either endovascular treatment (with MERCI Retriever or PENUMBRA System) or standard medical treatment within 8 h of anterior ischemic stroke symptom onset, and thus four subgroups based on perfusion results (penumbral versus non-penumbral) and type of treatment (standard medical versus endovascular) with 20–34 patients in each group were finally generated. According to the study outcomes, embolectomy was not found to be superior to standard care and the presence of a favorable penumbral pattern on neuroimaging could not identify patients who could benefit from endovascular therapy for AIS (18).

The latest three trials of endovascular treatment of acute stroke confirmed a strong association between the degree of successful revascularization and improved clinical outcomes and the importance of achieving rapid reperfusion. Moreover, they uncovered that intravenous and intra-arterial reperfusion therapies have similar safety profiles, including intracranial hemorrhage rates, and thus endovascular treatments were not associated with a greater benefit in acute stroke management compared to systemic thrombolysis (90).

However, both intra-arterial thrombolysis and thrombectomy may play a crucial role in providing treatment options for selected patients with persistent after intravenous thrombolysis large vessel occlusions (91). Current AHA/ASA guidelines suggest that intra-arterial fibrinolysis is beneficial for treatment of carefully selected patients with major MCA occlusion of <6 h duration who are not otherwise candidates for intravenous rt-PA (Class I; Level of Evidence B) and that rescue revascularization (with intra-arterial fibrinolysis or mechanical thrombectomy) may be reasonable in patients with large-artery occlusion who have not responded to intravenous fibrinolysis (27).

In a recent cohort study of 536 consecutive acute stroke patients treated with endovascular therapy, high revascularization rates (73.9%) with low rates of symptomatic intracerebral hemorrhage (5.6%) were noted. Age, stroke severity, hypertension, atrial fibrillation, and onset-to-treatment time were found to be independent predictors of functional outcome after endovascular treatment (92). Careful patient selection is mandatory for patients over 80 years of age, since the chances of gaining functional independence after endovascular recanalization, despite good recanalization rates and reasonable rates of hemorrhage, seem to be limited (93). In a recent meta-analysis of available literature data, stenting and mechanical thrombectomy were found to be associated with higher recanalization rates and improved functional outcomes compared with IA thrombolysis in AIS patients with extracranial and/or intracranial internal carotid artery occlusion (94). Finally, with the introduction of modern stent retrievers in the last decade, mechanical thrombectomy with modern stent retrievers has become a safe, rapid, and effective procedure with high recanalization rates and low risk of procedure-related complications in patients with basilar artery obstruction, a severe neurological and life-threatening condition (95, 96).

## Conclusion

Intravenous alteplase remains the fastest and easiest way to initiate acute stroke reperfusion treatment, and should continue to be the first-line treatment for patients with AIS within 4.5 h from onset. The use of tenecteplase instead of tPA and the augmentation of systemic thrombolysis with ultrasound are both novel therapeutical modalities that may emerge as significant options in AIS treatment. Endovascular treatments for AIS are rapidly evolving due to technological advances in catheter-based interventions and are currently emphasizing speed in order to result in timely restoration of perfusion of still-salvageable, infarcted brain tissue, since delayed recanalization of proximal intracranial occlusions has not been associated with improved clinical outcomes. The two stent retrievers (SOLITAIRE FR and TREVO) that have recently been approved by FDA for the treatment of AIS due to large vessel occlusion achieve faster and higher recanalization rates resulting in better three-month clinical outcomes in comparison to the older generation thrombectomy (MERCI) or thromboaspiration (PENUMBRA) devices. The safety and efficacy of stent retrievers in comparison to standard AIS treatment (intravenous thrombolysis within 4.5 h from symptom onset; antiplatelet therapy >4.5 h from symptom onset) remains to be proved in ongoing phase III randomized controlled trials [SWIFT PRIME, EXTEND-IA, THERAPY, BASICS, REVASCAT (97)]. Comprehensive imaging protocols in AIS may enable better patient selection for endovascular interventions and for testing multi-modal combinatory strategies.

## Conflict of Interest Statement

The authors declare that the research was conducted in the absence of any commercial or financial relationships that could be construed as a potential conflict of interest.
